# Recent developments in multiple sclerosis therapeutics

**DOI:** 10.1186/1741-7015-7-74

**Published:** 2009-12-07

**Authors:** Rebecca I Spain, Michelle H Cameron, Dennis Bourdette

**Affiliations:** 1 Oregon Health & Science University, Department of Neurology, CR120, 3181 SW Sam Jackson Park Road, Portland, OR 97239, USA

## Abstract

Multiple sclerosis, the most common neurologic disorder of young adults, is traditionally considered to be an inflammatory, autoimmune, demyelinating disease of the central nervous system. Based on this understanding, the initial therapeutic strategies were directed at immune modulation and inflammation control. These approaches, including high-dose corticosteroids for acute relapses and long-term use of parenteral interferon-β, glatiramer acetate or natalizumab for disease modification, are at best moderately effective. Growing evidence supports that, while an inflammatory pathology characterizes the early relapsing stage of multiple sclerosis, neurodegenerative pathology dominates the later progressive stage of the disease. Multiple sclerosis disease-modifying therapies currently in development attempt to specifically target the underlying pathology at each stage of the disease, while avoiding frequent self-injection. These include a variety of oral medications and monoclonal antibodies to reduce inflammation in relapsing multiple sclerosis and agents intended to promote neuroprotection and neurorepair in progressive multiple sclerosis. Although newer therapies for relapsing MS have the potential to be more effective and easier to administer than current therapies, they also carry greater risks. Effective treatments for progressive multiple sclerosis are still being sought.

## Introduction

Multiple sclerosis (MS) is a chronic progressive disorder of the central nervous system (CNS). It is traditionally considered to be an inflammatory disorder characterized by episodic CNS demyelination. However, current understanding is that neurodegeneration dominates the progressive stages of the disease. This article summarizes the pathogenesis of MS, reviews approved MS therapies and discusses the proposed mechanisms and likely benefits and risks of new MS therapeutics.

## Disease pathogenesis and disease subtypes

MS presents in most people (80%) with clinical relapses characterized by fully or partially-reversible focal neurological deficits [1]. Relapsing-remitting MS (RRMS) is dominated by inflammation, oedema and the physiologic actions of cytokines [2]. Active inflammation of the brain and spinal cord is visualized as gadolinium enhancing white matter lesions on magnetic resonance imaging (MRI). After 10-20 years, or median age 39.1 years, about half of those with RRMS gradually accumulate irreversible neurologic deficits in the absence of clinical relapses or new white matter lesions by MRI [3]. This stage is known as secondary progressive MS (SPMS). The remaining 20% with progressive clinical deterioration from the onset of the disease have primary progressive MS (PPMS) [1]. PPMS and SPMS are thought to be dominated by axonal degeneration in the absence of overt inflammation [4] which is most likely a result of oxidative damage and/or increased susceptibility to injury caused by the loss of the myelin sheath.

## Current MS therapeutics

Clinically significant acute MS relapses are usually treated with high-dose, short-term, intravenous corticosteroids (methylprednisolone 1 g/day for 3-5 days). This shortens relapse duration but does not improve the degree of recovery or the long-term course of the disease [5-7]. Disease-modifying therapies (DMT) for MS alter the course of the disease. They lower the clinical relapse rate, extend the time to next relapse and reduce the accumulation of new lesions on MRI, all of which are intended to decrease the long-term accumulation of disability. The first approved DMT for MS, subcutaneous interferon beta-1b (IFNβ-1b, marketed as Betaseron in the USA and as Betaferon in Europe), was approved by the US Food and Drug Administration (FDA) for RRMS in 1993. This was based on the pivotal placebo-controlled trial in which treated subjects had significantly lower annualised relapse rates and more subjects were relapse-free after 2 years [8]. IFNβ (interferon beta) acts as an anti-inflammatory and has several mechanisms of action, including a reduction in the production of pro-inflammatory IFNγ and TNFα, inhibition of T-cell activation and clonal expansion, modulation of cytokine and matrix metalloproteinase production and the release and inhibition of T-cell migration and entry into the CNS [9].

Since the release of IFNβ-1b, five other parenteral medications have been approved for the treatment of MS: IFNβ-1a (Avonex©, Biogen Idec, North Carolina, USA), IFNβ-1a (Rebif©, EMD Serono, Geneva, Switzerland), glatiramer acetate (GA, Copaxone©, Teva Pharmaceuticals, Petah Tikva, Israel), mitoxantrone (Novantrone©, OSI Pharmaceuticals, New York, USA) and natalizumab (Tysabri©, Biogen Idec, Massachusetts, USA). The IFN products are thought to all have similar mechanisms of action although they differ in the route of administration, rapidity of onset of action and risk of induction of neutralizing antibodies [10]. In contrast, GA - a synthetic copolymer of glutamic acid, lysine, alanine and tyrosine - is believed to activate Th2 regulatory cells in the periphery. These activated Th2 cells cross the blood brain barrier (BBB) and enter the CNS where they shift the immune response from pro-inflammatory to anti-inflammatory by secreting cytokines that down-regulate the inflammatory response and inhibit pro-inflammatory Th1 cells. Mitoxantrone is an antineoplastic agent that inhibits DNA and RNA synthesis of B and T-cells. While approved for the treatment of RRMS and SPMS [11-13], it has only shown a clear benefit for patients still experiencing relapses and developing new MRI lesions. Increasing recognition of short and long-term risks of cardiotoxicity, acute leukaemia and bone marrow suppression limit its use [14-16]. Natalizumab is the first monoclonal antibody (MAB) therapy approved for the treatment of MS. It binds to VLA-4 on the surface of leukocytes, preventing T-cells from crossing the BBB into the CNS [17]. Natalizumab was found to reduce MS relapses by 68% compared to placebo[18] but its use is limited by its association with the development of progressive multifocal leukoencephalopathy (24 patients at the time of this writing)[19], as well as melanoma[20]and primary CNS lymphoma[21] which are all probably due to an altered immune surveillance. In response, the FDA limited approval of natalizumab to patients failing other MS therapies and requires patients to be enrolled in a safety monitoring programme.

## Future directions

**Figure 1:**
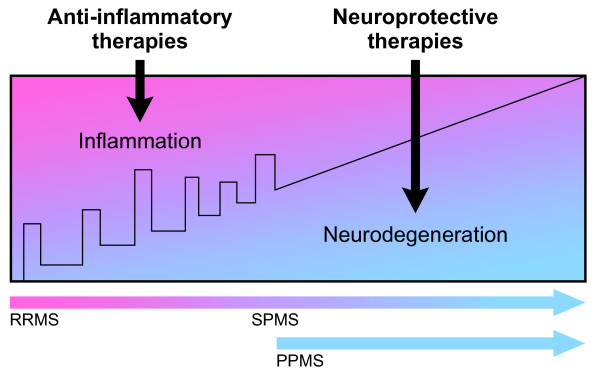
**Disease-modifying therapies in development target the pathology underlying the stage of multiple sclerosis (MS)**. Clinical course is indicated by the black line with stepwise increases in disability in early disease caused by relapses and, later, by gradual progression of disability in secondary progressive MS (SPMS) and from disease onset in primary progressive MS (PPMS). Anti-inflammatory therapies are effective in relapsing-remitting MS and SPMS when relapses are still present. Neuroprotective therapies target neurodegeneration in SPMS without relapses and PPMS.

DMT development for MS is an area of active research and many potential agents are in various phases of investigation. Most of these are oral medications or MABs that target specific aspects of inflammation in RRMS. Others are designed to have neuroprotective and neurorestorative effects in PPMS and SPMS (Figure 1).

### Oral anti-inflammatory agents for RRMS (Table 1)

**Table 1:** Oral anti-inflammatory agents for relapsing-remitting multiple sclerosis (RRMS).

Agent	Proposed mechanisms of action	Stage of development*
Fingolimod (FTY 720)	Binds sphingosine-1-phosphate on T cells preventing circulation and CNS entry	Phase III for RRMS
Cladribine	Selectively depletes B and T cells by causing DNA damage	Phase III for RRMS and CIS
Laquinimod	Shifts T cells from TH1 (pro-inflammatory) to TH2 (anti-inflammatory)	Phase III
Teriflunomide	Shifts T cells from TH1 (pro-inflammatory) to TH2 (anti-inflammatory); Blocks pyrimidine and reproduction of rapidly dividing B and T cells	Phase III for RRMS and CIS
BG00012 (dimethyl fumarate)	Antioxidant	Phase III

*compiled from clinicaltrials.gov

CIS, clinically isolated syndrome; CNS, central nervous system

Fingolimod (FTY720), a small molecule derived from a fungus and chemically related to sphingosine, causes internalization of sphingosine-1-phosphate (S1P) receptors on lymphocytes, thereby blocking their egress from lymph nodes and thymus. The reversible lymphocytopaenia prevents activated T cells from crossing the BBB and causing inflammation [22]. Fingolimod may also have direct effects via the S1P receptors present on all CNS cells types. A phase II clinical trial in RRMS found that fingolimod significantly reduced gadolinium-enhancing lesions and relapse rates compared to placebo [23] and a second trial demonstrated fingolimod's superiority to IFNβ-1a [24]. A European phase III clinical trial of fingolimod for RRMS has been completed, and a USA phase III trial is underway http://www.clinicaltrials.gov. Frequent adverse events associated with fingolimod include nasopharyngitis, dyspnea, headache and nausea. Rare serious adverse events in the trials were skin cancers and two deaths, one each from herpes encephalitis and disseminated varicella, all suggesting an inadequate immune surveillance.

The antimetabolite cladribine, an adenosine analogue, incorporates into DNA and causes the death of rapidly proliferating inflammatory B and T cells, thus resulting in selective and long-lasting lymphocyte depletion. Approved as a therapy for use in hairy-cell leukaemia, early trials indicated that cladribine was effective for RRMS but not SPMS [25,26]. A recent phase III trial in RRMS (CLARITY) demonstrated a significant benefit of cladribine over placebo with a reduction in annualized relapse rate of over 50% at 96 weeks [27]. Based on this trial, the manufacturers are applying for FDA registration for cladribine. Cladribine has an appealing short administration schedule and is generally well-tolerated, but there were some serious adverse events in the trial including herpes zoster in 2% of subjects, three cancers and four deaths in the cladribine groups [27].

There are three oral agents in phase III trials in RRMS, laquinimod, teriflunomide and BG00012. Laquinimode and teriflunomide are thought to work in part by shifting the T-cell balance from pro-inflammatory Th1 cells to anti-inflammatory Th2 cells. Laquinimod is a chemically and pharmacologically distinct derivative of the drug roquinimex, the study of which in RRMS was halted due to significant pulmonary and cardiac toxicity [28]. A 24 week phase II trial of laquinimod demonstrated a significant reduction of gadolinium-enhancing lesions on MRI over placebo [29]. Based on these promising early results, two phase III trials are underway in patients with RRMS. Teriflunomide is the active metabolite of leflunomide, a drug used in rheumatoid arthritis. It also inhibits dihydro-orotate dehydrogenase, the enzyme necessary for *de novo *synthesis of pyrimidine, thus reducing activated B and T cell proliferation [30]. A promising phase II trial [31] has led to phase III placebo-controlled trials of teriflunomide RRMS and clinically isolated syndrome (CIS). Need for pre-treatment with cholestyramine or activated charcoal and the frequent association of hypertension, alopecia and rash could potentially limit its use. BG00012 probably affects MS primarily through its anti-oxidant effects. It is an oral formulation of dimethyl fumarate, a topical agent used to treat psoriasis. Promising early studies [32] led to a phase III trial now underway.

Based on the observation that there is a reduction in MS relapses during pregnancy, a condition associated with high levels of and progesterone, it is thought that or progesterone may benefit patients with MS and progesterone have potent anti-inflammatory effects and may also provide neuroprotective benefits by increasing oligodendrocyte precursor cell number[33] and promoting oligodendrocyte process formation [34]. Phase III trials of oral estriol in conjunction with glatiramer acetate in RRMS are ongoing.

### Monoclonal antibodies for RRMS (Table 2)

**Table 2:** Monoclonal antibodies for relapsing-remitting multiple sclerosis (RRMS)

Agent	Proposed mechanisms of action	Stage of development*
Alemtuzamab	Leukocyte depletion; Depletes CD52 B and T cell populations, monocytes, macrophages and eosinophils	Phase III for RRMS
Daclizumab	Leukocyte depletion; Binds cell-surface receptor IL2; reduces T cell proliferation and activation	Phase II for RRMS
Rituximab	B cell-directed therapy; Depletes CD20 B cell population	Phase III for RRMS

*Compiled from http://www.clinicaltrials.gov

Alemtuzumab targets the CD52 antigen present on T cells, B cells, monocytes, macrophages and eosinophils, but not stem cells, and causes reversible leukocyte depletion. Alemtuzumab is approved for chronic lymphocytic leukaemia and showed promise in early trials for RRMS [35] but not SPMS [36]. Patients with RRMS treated with alemtuzumab compared to IFNβ-1a had significantly lower risk for relapse (75%) and reduction in sustained disability (65%) over 2 years [35]. Safety concerns included three cases of immune thrombocytopenic purpura with one fatality, Grave's disease, autoimmune anaemias and neutropenias and Guillain-Barre syndrome. Phase III trials are ongoing. Daclizumab depletes leukocytes by binding to the α-chain of the IL-2 receptor (CD25) required for T cell proliferation and activation. Daclizumab, approved for treatment of renal transplant rejection, appears to stabilize disease in RRMS patients who have failed IFN therapy [37,38] and is in Phase II clinical trials for RRMS. Adverse events associated with daclizumab include thromboses, lymphoproliferative disorders, and infections. Rituximab, a MAB that depletes CD20+ B cells, is an approved therapy for haematological malignancies, rheumatoid arthritis and thrombocytopenic purpura. B-cell dependent mechanisms such as antigen presentation, antibody secretion and demyelination, are increasingly implicated in the pathogenesis of MS [39,40]. A phase II trial [41] and case reports [42] show promise for its use in RRMS [43]. Adverse effects of rituximab include progressive multifocal leukoencephalopathy, pancytopenias, Stevens-Johnson syndrome and rare infections.

### Neuroprotective and neurorestorative agents for PPMS and SPMS

Thus far, all of the anti-inflammatory therapies that are effective in RRMS have had minimal or no effect in controlling progressive MS. There is a growing belief that SPMS and PPMS will not respond to anti-inflammatory therapies and that neuroprotective and neurorestorative therapies that affect neuronal integrity will be required for progressive MS. A variety of existing and novel approaches are under investigation as neuroprotective and neurorestorative therapies in progressive as well as relapsing MS (Table 3). Therapeutic strategies include protecting demyelinated axons as well as oligodendrocytes (the CNS myelin-producing cells) from oxidative injury, promoting neuronal remyelination and restoring neuronal growth and function with neurotrophic factors [44]. Lipoic acid, a fatty acid present in certain foods and available as an oral supplement, may protect oligodendrocytes by antioxidant mechanisms and effects on microglia. Lipoic acid is well tolerated [45] and phase II trials of this compound in RRMS and SPMS are being planned. Drugs that block glutamate receptors present on demyelinated axons may prevent oxidative injury [46]. Riluzole, an oral glutamate NMDA receptor antagonist approved for use in amyotrophic lateral sclerosis [47], is being evaluated in conjunction with IFNβ-1a in a phase II trial for early MS. Another strategy for neuroprotection in MS is selective sodium channel blockade with antiepileptic medications including phenytoin, topiramate and lamotrigine [48,49]. A recent phase II trial of lamotrigine in SPMS, however, failed to meet its primary endpoint of reducing the rate of central cerebral volume loss [50,51].

**Table 3:** Oral and parenteral neuroprotective and neurorestorative agents for primary progressive multiple sclerosis (MS) and secondary progressive MS.

Strategy	Drug class	Agents *
Protection of oligodendrocytes/axons from oxidative injury	Antioxidants	*Lipoic acid*
Protection of demyelinated axons from injury	Glutamate receptor antagonists	
	NMDA:	*Riluzole*
	AMPA/Kainate:	NBQX
		GYKI-52466
	Sodium channel blockers	*Phenytoin* *Topiramate* *Lamotrigine*
Promotion of remyelination	Stem cells	Embryonic, autologous
	LINGO-1	Anti-LINGO-1 antibodies
	Pregnancy hormones	*Estriol*
Neurotrophic factors to help restore neuronal function	Neurotrophic factors	
	Glial-derived:	GDNF, IGF, CNTF, neurturin, artemin, persephin
	AMPA/Kainate:	NGF, BDNF, NT3/4

*oral agents are indicated in italics

AMPA, α-amino-3-hydroxyl-5-methyl-4-isoxazole-propionate; BDNF, brain-derived neurotrophic factor; CNTF, ciliary neurotrophic factor; GDNF, glial cell line-derived neurotrophic factor; GYKI-52466, a 2, 3-benzodiazepine; IGF, insulin-like growth factor; LINGO-1, leucine rich repeat and Ig domain-containing, Nogo Receptor-interacting protein 1; NBXX, 2, 3-dihydroxy-6-nitro-7-sulfamoyl-benzo[f]quinoxaline-2,3-dione; NMDA, N-methyl-D-aspartic acid; NGF, nerve growth factor; NT3/4, neurotrophin 3 and 4.

Trials of stem cell transplantation, with the goal of repopulating oligodendrocytes, are underway in people with MS. Remyelination may also be promoted by blocking leucine rich repeat and Ig domain-containing, Nogo Receptor-interacting protein 1 (LINGO-1), a protein on the surface of neurons that inhibits differentiation of precursor oligodendrocytes into mature cells. Antibody blockade of LINGO-1 has shown promise in an animal model of MS [51]. Neurotrophins are protein factors produced by CNS cells that support neuronal growth, survival and differentiation [52]. In MS, secretion of the neurotrophin brain-derived neurotrophic factor (BDNF) is low and dysregulated [53] and BDNF is therefore also being considered as a therapeutic target.

## Conclusion

Current MS therapeutics are moderately effective for modifying disease during its relapsing-remitting phase. There are a number of oral and parenteral agents that target inflammation in development and several are likely to be approved for treatment of RRMS within the next few years. These therapies will likely more effectively control RRMS but will also carry greater known and, as yet, unknown safety risks. These risks and benefits will have to be weighed carefully against the efficacy and proven safety of the IFNs and GA. Furthermore, none of the anti-inflammatory therapies currently in late stage of development are likely to benefit patients with SPMS and PPMS. Development of effective neuroprotective and neurorestorative therapies are needed in order to benefit patients with progressive MS.

## Abbreviations

BBB: blood brain barrier; BDNF: brain-derived neurotrophic; CIS: clinically isolated syndrome; CNS: central nervous system; DMT: disease-modifying therapy; FDA: US Food and Drug Administration; GA: glatiramer acetate; IFN: interferon; LINGO-1: leucine rich repeat and Ig domain-containing, Nogo Receptor-interacting protein 1; MAB: monoclonal antibody; MRI: magnetic resonance imaging; MS: multiple sclerosis; PPMS: primary progressive MS; RRMS: relapsing-remitting MS; SIP: sphingosine-1-phosphate; SPMS: secondary progressive MS.

## Competing interests

RIS has no competing interests. MHC has received honoraria for speaking from Teva Neuroscience. DB has research grants from the National Institutes of Health, the Department of Veterans Affairs and the National Multiple Sclerosis Society. DB has also received honoraria for speaking/consulting or unrestricted educational grants, from Teva Neuroscience, Biogen Idec, EMD Serono and Bayer USA.

## Authors' contributions

RIS and MHC contributed equally to the paper. All authors were involved in the drafting of the manuscript and revision for important intellectual content. All authors read and approved the final manuscript and have given their approval of final published version.

## Pre-publication history

The pre-publication history for this paper can be accessed here:

http://www.biomedcentral.com/1741-7015/7/74/prepub
